# Development of a universal cutting guide for raising deep circumflex iliac artery flaps

**DOI:** 10.1007/s11548-024-03144-9

**Published:** 2024-04-27

**Authors:** Florian Peters, Stefan Raith, Anna Bock, Kristian Kniha, Stephan Christian Möhlhenrich, Marius Heitzer, Frank Hölzle, Ali Modabber

**Affiliations:** 1 Department of Oral, Maxillofacial and Facial Plastic Surgery, University Hospital RWTH Aachen, Pauwelsstr. 30, 52074 Aachen, Germany; 2 Department of Orthodontics, University Witten/Herdecke, Witten, Germany

**Keywords:** DCIA, DCIA flap, VSP, CAM, Oral cancer, Jaw reconstruction

## Abstract

**Purpose:**

The deep circumflex iliac crest flap (DCIA) is used for the reconstruction of the jaw. For fitting of the transplant by computer-aided planning (CAD), a computerized tomography (CT) of the jaw and the pelvis is necessary. Ready-made cutting guides save a pelvic CT and healthcare resources while maintaining the advantages of the CAD planning.

**Methods:**

A total of 2000 CTs of the pelvis were divided into groups of 500 by sex and age (≤ 45 and > 45 years). Three-dimensional (3D) pelvis models were aligned and averaged. Cutting guides were designed on the averaged pelvis for each group and an overall averaged pelvis. The cutting guides and 50 randomly selected iliac crests (10 from each group and 10 from the whole collective) were 3D printed. The appropriate cutting guide was mounted to the iliac crest and a cone beam CT was performed. The thickness of the space between the iliac crest and the cutting guide was evaluated.

**Results:**

Overall the mean thickness of the space was 2.137 mm and the mean volume of the space was 4513 mm^3^. The measured values were significantly different between the different groups. The overall averaged group had not the greatest volume, maximum thickness and mean thickness of the space.

**Conclusion:**

Ready-made cutting guides for the DCIA flap fit to the iliac crest and make quick and accurate flap raising possible while radiation dose and resources can be saved. The cutting guides fit sufficient to the iliac crest and should keep the advantages of a standard CAD planning.

## Introduction

Squamous cell carcinoma of the head and neck (HNSCC) is a common form of cancer with an increasing incidence [1]. For small HNSCC in the oral cavity without metastasis in the lymphatic nodes, the surgical resection can achieve a cure rate of up to 80% [2]. Even for larger tumors, surgical resection is chosen as a treatment in combination with radiation and systemic therapy. In advanced cases, the ablation of the jaw bone is necessary in curative treatment. However, large benign tumors, extensive necrosis of the jaw or trauma can also lead to the loss of a large part of the jaw. This leaves the patients with reduced quality of life [3].

After ablative surgery of the jaw, the bony reconstruction using microvascular bone flaps is the gold standard in the literature [4]. The deep circumflex iliac crest (DCIA) flap is commonly used for the reconstruction of the jaw and following dental prosthetic rehabilitation using enosseous dental implants [5, 6].

The pelvis is a central bone in the human body. It is crucial in human locomotion and for obstetrics [7]. It is a bone with huge dimorphism and growth between males and females [8]. This results in many different morphologies of the pelvis and consequently the iliac crest. Currently, for exact placement and size of the DCIA flap computer-aided planning (CAD) is regularly used prior to surgery [9].

For CAD planning, a computerized tomography (CT) of the jaw and the pelvis is necessary [10, 11]. The CT of the jaw is necessary for measuring the bony defect and the placement of the later DCIA flap. The CT of the pelvis is necessary for the design of the transplant and the cutting guides for harvesting the DCIA flap. However, the price for the higher accuracy achievable with CAD planning is a high radiation dose for the patient. The dose of a head CT is 1–2 mSv and the dose of a pelvis CT accumulates to 3–4 mSv [12]. If the advantages of the CAD planning could be obtained without the necessity of a pelvis CT, the patients’ dose could be reduced about 75%. In addition, the cost of computerized-planning is high, making it difficult to use in societies with less well-funded healthcare systems. A ready-made cutting guide with predefined outlines that fits to the donor bone and can easily be customized in its’ extensions could reduce the cost significantly. No standard CAD planning is carried out, as the cutting guides are not planned on the individual iliac crest but a universal guide is used to assist raising the DCIA flap. Nevertheless, the extent of the cutting guide will be digitally adjusted preoperatively to the defect of the jaw to be reconstructed.

The anatomy of the nourishing vessels of the DCIA flap is well known and recent publications show no severe stenosis by atherosclerosis [13]. Therefore, a CT scan is only necessary to obtain the anatomy of the iliac crest to create the cutting guide.

In every day clinical practice, numerous CT scans of the pelvis are performed for several reasons. Due to legal reasons, the pictures have to be archived and create a huge amount of data that can be used for a secondary purpose. Following the trend of big data in medicine, the data could enable to create average models of the pelvis for designing cutting guides for the iliac crest that fit to nearly every iliac crest.

Therefore, it was hypothesized that a ready-made cutting guide for the iliac crest could be developed from the data, making a pelvic CT unnecessary before DCIA flap harvest.

## Materials and methods

After institutional approval (EK 260/20) of the local ethics committee, the local picture archiving and communication system was searched for CT examinations including the complete pelvis. Exclusion criteria were as follows: artifacts, fractures, implants, obvious deformities. A total number of 2000 CT scans were downloaded. From these 500 CT scans were grouped in each group divided for gender and age. For females and males, there were two groups each. One group consisted of patients from 18 to 45 years (YF: females ≤ 45 years; YM: males ≤ 45 years) and the other consisted of patients from 46 to 99 years (OF: females > 45 years; OM: males > 45 years). In addition, there was a universal group (UNIV) in which all 2000 CT scans were. The division into groups according to sex is based on the sexual dimorphism of the pelvis [14]. A division by age was necessary because of the different growth patterns of the pelvis before and after menopause [8]. The data was saved as digital imaging and communications in medicine (DICOM) files.

### Segmentation

The DICOM files of each examination were imported into ProPlan CMF version 3.0.1 (Materialise, Leuven, Belgium). In this software, the DICOM images were segmented and converted into three-dimensional (3D) models of the pelvis. Therefore, the spine was cut above the Os sacrum and the femur was removed from the pelvis model. After segmentation, the model was saved as a standard tessellation language (STL) file.

### Definition of a generalizable topology

**Fig. 1:**
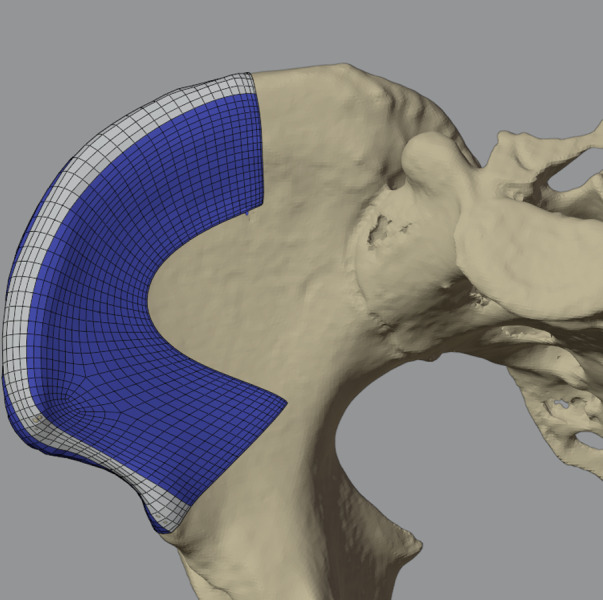
The quadrilateral mesh used for averaging morphed onto the region of interest on the iliac crest

As all geometrical models stored in STL files are arbitrarily meshed, a direct geometrical relation that allows statistical evaluation is not possible. Hence, it was necessary to define a mesh topology that can be individualized to represent the different geometrical shapes of the bones. To avoid geometrical errors due to mesh deformation, quadrilaterals were chosen over triangular meshes, as this allows a consistent topological flow, i.e., following anatomical features, such as the top of the iliac crest, and is more robust toward deformations (Fig. 1).

The topologies were designed to respect salient anatomical structures and to cover the portions of the iliac crest that are relevant for transplant harvesting in reconstructive surgery [15]. To individualize the standard topology in order to represent accurately the shape of each individual pelvic bone surface, a morphing based on guiding landmarks and a cascade of subsequent projection and mesh smoothing operations was performed as implemented in the software Blender version 2.7.8 (Blender Foundation, Amsterdam, Netherlands) and previously reported [16]

### Alignment and averaging

Each of the 2000 pelvic models was imported into Blender version 2.7.8 (Blender Foundation, Amsterdam, Netherlands). First, a coarse alignment of the models was performed manually to place them in the center of the global coordinate system and give them matching orientations by turning and tilting until both iliac crests were at the same height and both spinae iliaca anterior superior were facing to the front. This coarse alignment was required to standardize the subsequent steps of generating topologically standardized meshes, as described in the previous section. By the use of a custom in-house developed plugin for the software Blender, anatomical landmarks were placed automatically in relation to salient geometrical features of the pelvic bone models. These landmarks were used to guide the geometry of the standardized topology. To allow for an individual fine adjustment, it was possible to modify the positions of these guiding landmarks. This step was performed by one single, specifically trained operator after defining standardized criteria. The geometrical data of the topologically standardized meshes was stored in a custom file format to allow subsequent statistical averaging.

In the scope of geometrical averaging, all data was loaded and aligned to a common coordinate system by applying the generalized Procrustes analysis (GPA) [17], while omitting scale correction. By doing so, averages were calculated for the whole population.

### Cutting guide design and 3D printing

**Fig. 2:**
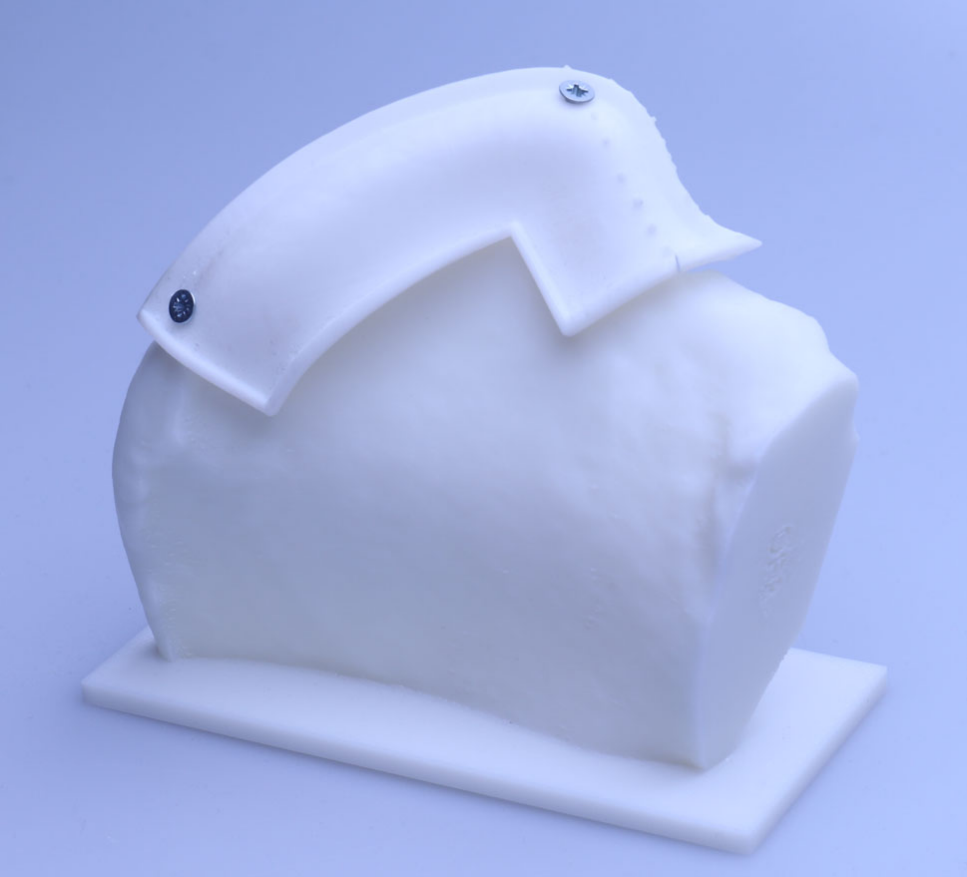
Ready-made cutting guide fixed to 3D printed iliac crest model

The cutting guides were designed on the 3D model of the averaged pelvis. One cutting guide was designed for each group and one cutting guide was designed on the 3D model of the pelvis, which was averaged from all models (UNIV: overall averaged cutting guide group). The guides were designed with hockey stick layout and an offset of 1 mm to the pelvis. The width of the cutting guides was 30 mm measured from the iliac crest to the caudal border and 30 mm from the spina iliaca anterior superior to the dorsal border (Fig. 2). This design was chosen because it represents the maximum bone portion nourished by the circumflex ilium profunda artery, which is the nourishing vessel. The vessel reaches the Spina iliaca anterior superior at the medial side of the iliac crest [18]. It continues dorsally on the inner side and gives off small branches to the bone. After about 6 cm it crosses the iliac crest, and from there it nourishes the bone [19]. The total length was 80 mm. Also, if bone elevation to the sacroiliac joint is possible, this length is sufficient for most mandibular reconstructions and keeps the integrity of the sacroiliac joint.

From each of the four groups, 10 models were randomly selected and 3D printed. For printing a Stratasys, Fortus 450 mc printer (Stratasys, Eden Prairie, MN, USA) was used. The models were printed with Acrylnitril–Butadien–Styrol copolymer (ABS). Respectively, 10 cutting guides of each group and 10 overall averaged cutting guides were 3D printed via selective laser sintering out of Polyamid 12 (PA12).

### Accuracy test

The printed cutting guide was fixed to the printed iliac crest of the matching group with two screws. To do this, a sliding hole was drilled through the template and the screws were tightened manually without creating tension on the guide. This prevents deformation of the guide. Then, the models were scanned with the cone beam CT Galileos Comfort Plus (Sirona Dental Systems GmbH, Wals, Austria) with 98 kV and 30 mAS. The resulting 3D scan was imported into Mimics inPrint version 3.0.0.249 (Materialise, Leuven, Belgium). The gap between the outer surface of the iliac crest and the cutting guide was segmented and saved a STL file.

**Fig. 3:**
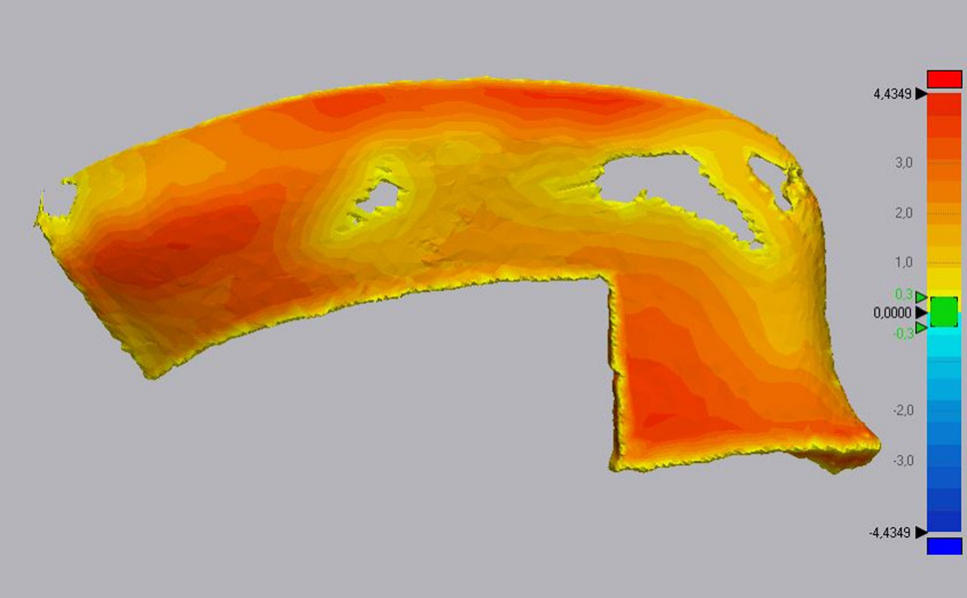
Heatmap of mean thickness measured on the space between pelvis model and cutting guide

Evaluation of the models was performed in Geomagic Control X (3D Systems Corporation, Rock Hill, SC, USA). In the software, the thickness of the model was measured (Fig. 3). The mean thickness, and the maximum thickness as well as the volume of the space between the cutting guide and the model of the pelvis were recorded.

### Statistical analysis

Statistical analysis was performed using Prism GraphPad version 9 (GraphPad Software Inc., San Diego, California, USA). A Mann–Whitney *U* test was performed, and the level of significance was set as *p* ≤ 0.05. All data was expressed as mean values ± standard deviation.

## Results

The measured values for the minimum and maximum thickness and the volume of the space between the cutting guide and the pelvic model are shown in Table 1.

**Table 1:** Measured results for mean thickness, maximum thickness and volume of the air layer

	UNIV	OF	OM	YF	YM
Mean thickness (mm)	2.267 ± 0.6055	2.425 ± 0.8716	2.757 ± 1.437	1.695 ± 0.8477	1.543 ± 0.7045
Maximum thickness (mm)	6.318 ± 1.459	6.155 ± 1.993	6.735 ± 2.519	5.478 ± 1.89	4.093 ± 1.156
Volume of air layer (mm^3^)	4807 ± 1346	4299 ± 1579	6130 ± 2931	4354 ± 2111	2977 ± 1453

*UNIV* Universal cutting guide group; *OF* females > 45 years cutting guide group; *OM* males > 45 years cutting guide group; *YF* females ≤ 45 years cutting guide group; *YM* males ≤ 45 years cutting guide group

**Fig. 4:**
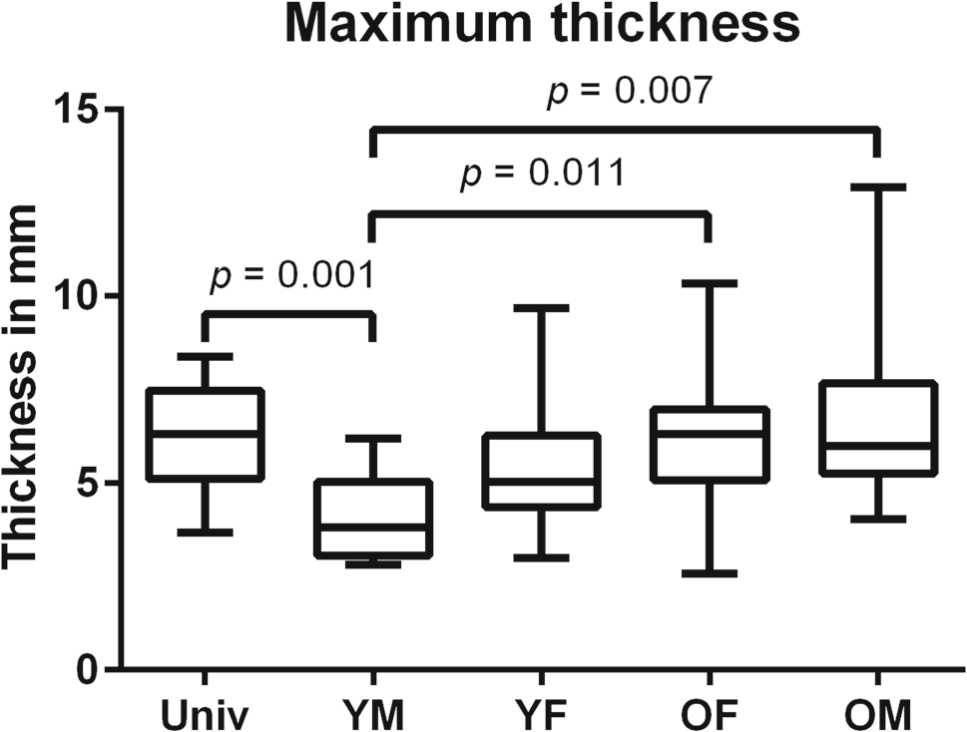
Boxplot of the maximum thickness of the space between the pelvis model and the gutting guide. *UNIV* Universal cutting guide group; *OF* females > 45 years cutting guide group; *OM* males > 45 years cutting guide group; *YF* females ≤ 45 years cutting guide group; *YM* males ≤ 45 years cutting guide group

**Fig. 5:**
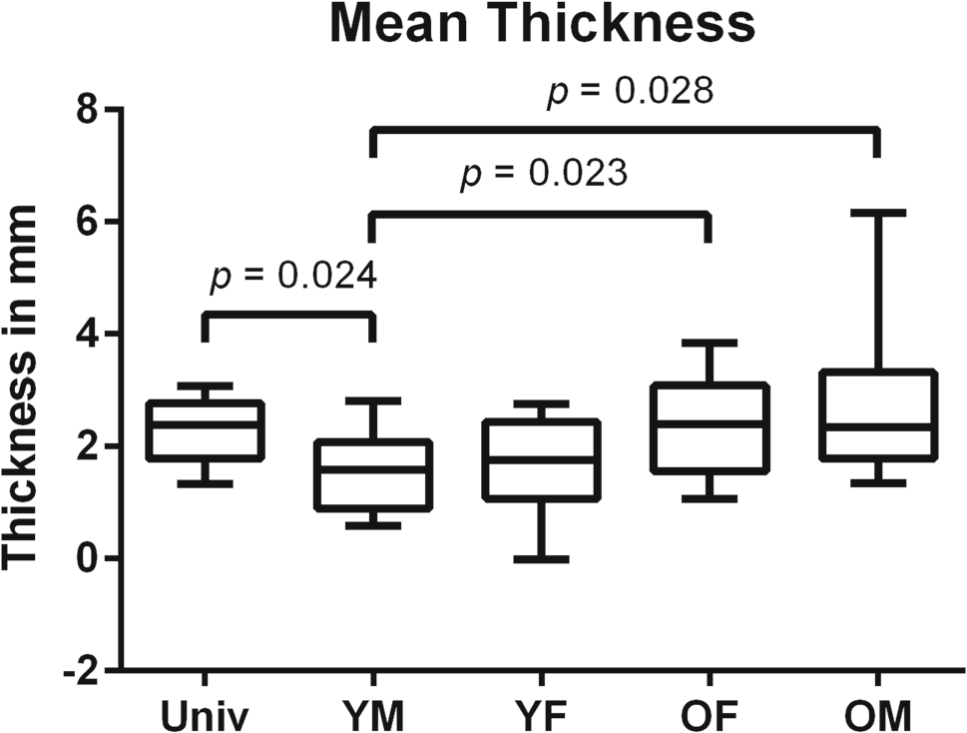
Boxplot of the mean thickness of the space between the pelvis model and the gutting guide. *UNIV* Universal cutting guide group; *OF* females > 45 years cutting guide group; *OM* males > 45 years cutting guide group; *YF* females ≤ 45 years cutting guide group; *YM* males ≤ 45 years cutting guide group

**Fig. 6:**
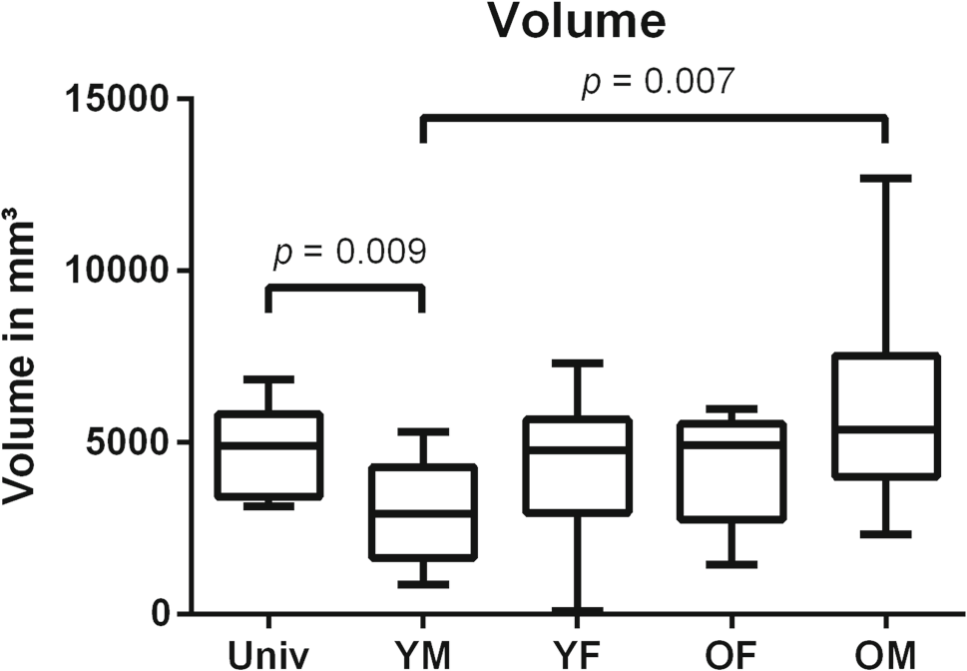
Boxplot of the volume of the space between the pelvis model and the gutting guide. *UNIV* Universal cutting guide group; *OF* females > 45 years cutting guide group; *OM* males > 45 years cutting guide group; *YF* females ≤ 45 years cutting guide group; *YM* males ≤ 45 years cutting guide group

In all measured values, the YM group reached significantly lower values than the UNIV and the OM group (Figs. 4, 5, 6). For the maximum and the mean thickness, the values of the YM group were significantly lower than in the OF group (Figs. 4, 5). All other differences between the groups were not significant. The group YM reached the lowest values for each measured category, while the UNIV group did neither reach the greatest thickness nor the highest volume (Table 1).

## Discussion

The microvascular reconstruction with a DCIA flap offers enough bone for dental rehabilitation via enossal dental implants and an excellent preformed shape for reconstruction of the mandibular angle [20–22]. Before CAD planning was the gold standard for preoperative planning of the DCIA flap transfer [23], the result was highly dependent from the experience and skill of the surgeon. Using CAD planning enables the preoperative simulation of different variants and conditions [24, 25]. The first hurdle in conventional raising of a DCIA flap is determining the necessary dimensions of the graft. To do this, a paper sheet or a metal plate is commonly adapted to the defect as a template and then transferred to the iliac crest using the template. The surgeon has to oversize the template because he can grind away excess bone freehand. However, if the transplant is too small, the missing bone can only be added with difficulty. Compared to the conventional raising of the flap, the CAD planning of microvascular bone flaps for reconstruction of the mandible led to a shortened ischemic time of the transplant [26] and less time for shaping the transplant [27]. Additionally, the supernatant of bone harvested from the pelvis is smaller than harvesting the DCIA with conventional techniques [27]. These advantages have made preoperative CAD planning of microvascular bone flaps the gold standard. The cutting guides developed in this study are intended to offer the same advantages as complete CAD planning, without the need for a pelvic CT. This is achieved by preoperatively adapting the universal cutting guide to the defect of the jaw. The dimensions of the cutting guide are digitally adjusted to the size of the defect and then the universal cutting guide is printed. This means that the DCIA flap can be raised and there is no excess bone but, above all, there is no undersized flap. Due to the precise lifting and the lack of adjustment of the DCIA flap after raising, the cutting guides presented here can be used to keep the excess of raised iliac crest small and reduce the ischemic time compared to the conventional method of flap raising.

A disadvantage that comes with preoperative CAD planning is the necessity of 3D images of the pelvis and the mandible. While the images of the mandible are commonly already available due to the underlying disease, the 3D images of the pelvis must be made exclusively for CAD planning. Unlike the free fibula flap, the vascular anatomy of the DCIA is nearly constant and so angiography is unnecessary [15, 28]. The dose of a CT of the pelvis is around 3–4 mSv [12]. This dose is comparable to the whole natural background radiation dose of a citizen in the USA for a whole year with 3.11 mSv [29]. HNSCC patients receive additional CT scans during follow-up but only of the head and neck area [30]. Without the pelvic CT scan, the cumulative dose for these patients could be reduced.

A completely new approach in medicine is the use of big data [31, 32]. Accessing big data opens up the possibility to improve medical processes. The processing of big data becomes possible with increasing computing capacity. Therefore, it was the aim of this study to prove that the usage of a large dataset of pelvic anatomy could make a CT scan of the pelvis prior CAD planning of the DCIA flap obsolete.

Since dose avoidance is a principle of radiation protection, any avoidable exposure to ionizing radiation should be avoided. Therefore, the aim of this study was to use CT scans already obtained for other reasons to calculate a standard geometry of the iliac crest and design cutting guides on this average geometry. This could make the preoperative CT scan of the pelvis obsolete and save a radiation dose of 3–4 mSv for the patient. Additionally, expensive and scarce resources could be saved in the health system and reduce the expenses for the patient.

The weight of the intestines is transferred to the legs and children are delivered through the female pelvis, leading to a sexual dimorphism between male and females pelvises [14]. The morphological dimorphism is not equally pronounced at every structure of the pelvis. Current literature describes the sexual dimorphism at the iliac crest, which is used for DCIA harvesting, as moderate [33]. Furthermore, because of the different hormonal development of males and females the morphology of the pelvis changes depending not only on the sex but also the age. The female pelvis develops in a different pattern in younger years than after the menopause [8]. To address these differences, a separation of males and females as well as a separation in two separate groups of age was necessary for creating the best fitting stock cutting guide for the DCIA flap. Additionally, a universal cutting guide group (Univ) was created. The Univ group contained all pelvis models for averaging. This group was used as a control group to check whether the gender and age split actually resulted in a better fit of the cutting guides. The CT data used for averaging was obtained in daily routine and retrospectively accessed. Therefore, the ethnicity and race as well as the exact body habitus could not be considered for separation of the data into different groups.

The study was deliberately not conducted purely digitally. Due to the printing of the anatomical models, a printer error of ± 0.127 mm was accepted [34]. However, printing the guides would also be necessary when using them on patients. Therefore, the additional error due to the renewed digitization of the models using CBCT was also accepted. A purely digital comparison of the fit would disregard these processes, which are necessary for use on the patient.

The fitting of the cutting guides to the pelvis was examined by measuring the space between the cutting guide and the iliac crest which was filled with air. The incongruence between the ready-made cutting guide developed in this study and the actual iliac crest of the patient should not result in an error that makes the jaw reconstruction with the DCIA flap impossible. The space had a mean thickness of 2.267 ± 0.6055 mm and a maximum thickness of 6.318 ± 1.459 mm in the UNIV group. Even a gap of 6.5 mm between the guide with its flange and the bone enables the surgeon to securely saw the DCIA transplant at the defined position.

The allocation of the pelvis CTs into different groups led to a significantly reduced mean and maximum thickness of the space as well as their volume for the YM group compared to the UNIV group (*p* = 0.024; *p* = 0.001; *p* = 0.009). Additionally, the results of the YM group were significantly lower in mean and maximum thickness compared to the OF and OM group (*p* values see Figs. 4, 5, 6) while there was no significant difference for the mean and maximum thickness and the volume between the UNIV group and the YF, OF and OM group. This leads to the assumption that the sexual dimorphism of the pelvis is not that distinct at the iliac crest. Furthermore, the missing significant difference for the mean and maximum thickness as well as the volume between the groups YF and OF shows that the hormonal effect on the morphology of the iliac crest is negligible.

Exact data on how precise the cutting guides fit to the iliac crest is not available in the literature. However, the use of CAD planned cutting guides for osteotomies of free bone flaps should lead to an accurate transition of the virtual plan from the computer into the patient. Nevertheless, the use of 3D printed cutting guides implements an error in this transition [35, 36]. The error does not only result from the design or the existence of the sawing slots, or flanges [37], the secure and reproducible positioning of the guides on the bone is crucial as well [38, 39]. This results in deviations from the original planned position of the osteotomy to the actual position of 1.2 ± 1.0 mm to 2.00 ± 1.12 mm [35, 38]. A perfect transition from the virtual plan into the patient is not possible with this technique. The cutting guides designed in this study should achieve comparable accuracies for the position of the osteotomy as reported in the literature.

As described in the Material and Methods section, the cutting guides in this study were designed for the raising a hockey stick-shaped DCIA flap. However, the dimensions of the universal cutting guides can be modified to adapt the DCIA flap to the bone defect to be reconstructed. A reduction in the dimensions of the cutting guide would in most cases lead to a better fitting of the universal guide with the iliac crest, as in the cases tested in this study the edge of the cutting guide rested at some point. By removing this point, the entire guide can better adhere to the iliac crest. In cases where the edge of the cutting guide does not rest on the iliac crest and the size is reduced, the distance to the iliac crest cannot increase as the contact surface remains the same. A deterioration of the fit by adjusting the size of the guides to a defect is therefore not to be expected.

## Conclusion

Even though the anatomy of the iliac crest is highly variable, the large amount of data allows the development of a ready-made cutting guide for the DCIA. These can be adapted to the determined defect size of the mandible and allow the DCIA flap to be lifted quickly and accurately without the need for CT imaging of the pelvis. Thus, significant radiation dose can be saved while maintaining the surgical quality and improving the patient’s outcome.
